# Supplementary material for: Therapeutic Monoclonal Antibodies against Cancer: Present and Future
Source: Cells. 2023 Dec 14;12(24):2837. doi: 10.3390/cells12242837 (PMC10741644; doi:10.3390/cells12242837)
Supplement: Supplementary file 1 [file cells-12-02837-s001.zip › cells-2761262-supplementary.pdf]

cells

MDPI

Table S1. Description of commercially available therapeutic antibodies either approved or in late review stages and their molecular targets.

International Non-Proprietary Name	Indication First approved	Target	Heavy/light Chains/Specificity	First global approval (country, year) or in review	Conjugation/Payload	Fc Modifications
Atezolizumab	bladder cancer	PD-L12	IgG1/k/Mono	US, 2016		Remove Fc effector functions
Durvalumab	bladder cancer	PD-L12	IgG1/k/Mono	US, 2017		Remove Fc effector functions
[fam-] trastuzumab deruxtecan-nxki	breast cancer	HER2	IgG1/k/Mono	US, 2019	ADC[3]: DXd[5]	
Ado-trastuzumab emtansine	breast cancer	HER2	IgG1/k/Mono	US, 2013	ADC[3]: DM1[6]	
Margetuximab-cmkb	breast cancer	HER2	IgG1/k/Mono	US, 2020		Improved FcγRIIIa binding[15] Improved ADCC
Cipterbin[a]	breast cancer	HER2	IgG1/n.a.[1]/Mono	China, 2020		Enhanced ADCC/ADCP[16]
Pertuzumab	breast cancer	HER2	IgG1/k/Mono	US, 2012		
Trastuzumab	breast cancer	HER2	IgG1/k/Mono	US, 1998		
Trastuzumab duocarmazine	breast cancer	HER2	IgG1/k/Mono	in review	ADC[3]: Duocarmycin[7]	
Trastuzumab botidotin	breast cancer	HER2	IgG1/k/Mono	in review	ADC[3]: Duostatin-5[8]	
Sacituzumab govitecan-hziy	breast cancer: TNBC	TROP-2	IgG1/k/Mono	US, 2020	ADC[3]: SN38[9]	
Enlonstobart	cervical cancer	PD-12	IgG4/k/Mono	in review		
Cadonilimab	cervical cancer	PD-12, CTLA42	IgG1/k/Bi	China, 2022		Reduced Fc effector function
Socazolimab	cervical cancer	PD-L12	IgG1/l2/Mono	in review		
Tisotumab vedotin-tftv	cervical cancer	Tissue factor	IgG1/k/Mono	US, 2021	ADC[3]: MMAE[10]	
Omburtamab	CNS, neuroblastoma	B7-H3	mIgG1/k/Mono	in review	RIC[4]: Iodine-131	
Dinutuximab	CNS, neuroblastoma	GD2	IgG1/k/Mono	US, 2015		
Naxitamab-gqgk	CNS, neuroblastoma	GD2	IgG1/k/Mono	US, 2020		
Cetuximab	colon/colorectal cancer	EGFR	IgG1/k/Mono	Switzerland, 2003		
Panitumumab	colon/colorectal cancer	EGFR	IgG2/k/Mono	US, 2006		
Edrecolomab	colon/colorectal cancer	EpCAM	mIgG2a/k/Mono	Germany, 1995		
Bevacizumab	colon/colorectal cancer	VEGF-A	IgG1/k/Mono	US, 2004		
Dostarlimab	endometrial cancer	PD-12	IgG4/k/Mono	EU, 2021		Hinge stabilizing
Zolbetuximab	esophageal/gastric cancer	Claudin-18.2	IgG1/k/Mono	in review		
Disitamab vedotin	gastric cancer	HER2	IgG1/k/Mono	China, 2021	ADC[3]: MMAE[10]	
Ramucirumab	gastric cancer	VEGFR2	IgG1/k/Mono	US, 2014		

Cetuximab saratolacan	head & neck cancer	EGFR	IgG1/k/Mono	Japan, 2020	Photoimmunotherapy IRDye® 700DX	
Nimotuzumab	head & neck cancer	EGFR	IgG1/k/Mono	Cuba, 2002		
Emapalumab-lzsg	hemophagocytic lymphohistiocytosis	IFNg	IgG1/l/Mono	US, 2018		
Blinatumomab	leukemia: ALL	CD19, CD3	Tandem scFv/n.a./Bi	US, 2014		
Inotuzumab ozogamicin	leukemia: ALL	CD22	IgG4/k/Mono	US, 2017	ADC[3]: Calicheamicin[11]	Hinge stabilizing
Gemtuzumab ozogamicin	leukemia: AML	CD33	IgG4/k/Mono	US, 2000	ADC[3]: Calicheamicin[11]	Hinge stabilizing
Obinutuzumab	leukemia: CLL	CD20	IgG1/k/Mono	US, 2013		Improved FcγRIIIa binding/Improved ADCC
Ofatumumab	leukemia: CLL	CD20	IgG1/k/Mono	US, 2009		
Alemtuzumab	leukemia: CML	CD52	IgG1/k/Mono	US, 2001		
Moxetumomab pasudotox-tdfk	leukemia: HCL	CD22	dsFv fused with PE38 exotoxin//Mono	US, 2018	Immunoconjugate PE38	
Tremelimumab	liver cancer	CTLA-42	IgG2/k/Mono	US, 2022		
Ivonescimab	lung cancer	PD-12, VEGF	IgG1/k/Bi	in review		Removed Fc effector functions
Necitumumab	lung: non-small cell	EGFR	IgG1/k/Mono	US, 2015		
Racotumomab	lung: non-small cell	GM3	mIgG1/k/Mono	Cuba, 2013		
Sintilimab	lung: non-small cell	PD-12	IgG4/k/Mono	China, 2018		Hinge stabilizing
Sugemalimab	lung: non-small cell	PD-L12	IgG4/l/Mono	China, 2021		Hinge stabilization
Adebrelimab	lung: small cell	PD-L12	IgG4/k/Mono	China, 2023		Hinge stabilization; abrogated effector function
Benmelstobart	lung: small cell	PD-L12	IgG1/k/Mono	in review		Removed Fc effector functions
Loncastuximab tesirine	lymphoma: B cell	CD19	IgG1/k/Mono	US, 2021	ADC[3]: SG3199[12]	
Tafasitamab-cxix	lymphoma: B cell	CD19	IgG1/2 hybrid/k/Mono	US, 2020		Improved FcγRIIIa binding/Improved ADCC
Zuberitamab	lymphoma: B cell	CD20	IgG1/k/Mono	China, 2023		
Epcoritamab	lymphoma: B cell	CD20, CD3	IgG1; Hetero H/l, k/Bi	US, 2023		Chain pairing, Reduced Fc effector function
Odronextamab	lymphoma: B cell	CD20, CD3	IgG4 Hetero H, cL/k/Bi	in review		Fc-silencing and hinge-stabilization
Glofitamab	lymphoma: B cell	CD20, CD3e	IgG1; CrossMab; Fab-Fc(G1) x Fab-Fab-Fc/l, k/Bi	Canada, 2023		Chain pairing, Reduced Fc effector function
Polatuzumab vedotin-piiq	lymphoma: B cell	CD79b	IgG1/k/Mono	US, 2019	ADC[3]: MMAE[10]	
Mosunetuzumab	lymphoma: Folicular	CD20, CD3	IgG1; Hetero H, HL assembly/k/Bi	EU, 2022		Improved FcγRIIIa binding/Improved ADCC

Brentuximab vedotin	lymphoma: Hodgkin's	CD30	IgG1/k/Mono	US, 2011	ADC[3]: MMAE[10]	
Camrelizumab	lymphoma: Hodgkin's	PD-12	IgG4/k/Mono	China, 2019		Hinge stabilization
Zimberelimab	lymphoma: Hodgkin's	PD-12	IgG4/l/Mono	China, 2021		Hinge stabilization
Ibritumomab tiuxetan	lymphoma: non-Hodgkin's	CD20	mIgG1/k/Mono	US, 2002	RIC4: Yttrium-90	
Ripertamab	lymphoma: non-Hodgkin's	CD20	IgG1/k/Mono	China, 2022		
Rituximab	lymphoma: non-Hodgkin's	CD20	IgG1/k/Mono	US, 1997		
Tositumomab-I131	lymphoma: non-Hodgkin's	CD20	mIgG2a/l/Mono	US, 2003	RIC[4]: Iodine-131	
Catumaxomab	malignant ascites	EPCAM, CD3	mIgG2a/k and rIgG2b/λ Hybrid/k, l/Bi	EU, 2009		
Ipilimumab	melanoma	CTLA-42	IgG1/k/Mono	US, 2011		
Tebentafusp	melanoma	gp100, CD3	scFv fused with a TCR/n.a./Bi	US, 2022	Immunoconjugat e	
Relatlimab	melanoma	LAG-3	IgG4/k/Mono	US, 2022		Hinge stabilizing
Pembrolizumab	melanoma	PD-12	IgG4/k/Mono	US, 2014		Hinge stabilizing
Prolgolimab	melanoma	PD-12	IgG1/V-l, C-k/Mono	Russia, 2020		Reduced Fc effector function
Nivolumab	melanoma/lung: non-small cell	PD-12	IgG4/k/Mono	US, 2014		Hinge stabilizing
Amivantamab	lung: non-small cell-EGFRmut	EGFR, cMET	IgG1/k/Bi	US, 2021		Improved FcγRIIIa binding & ADCC; Controlled Fab arm exchange for bispecific generation
Retifanlimab	merkel cell carcinoma	PD-12	IgG4/k/Mono	US, 2023		Hinge stabilizing
Avelumab	merkel cell carcinoma	PD-L12	IgG1/l/Mono	US, 2017		
Pucotenlimab	microsatellite instability-high solid tumors	PD-12	IgG4/k/Mono	China, 2022		Hinge stabilzation, half-life extension
Serplulimab	microsatellite instability-high solid tumors	PD-12	IgG4/k/Mono	China, 2022		Hinge stabilization
Envafolimab	microsatellite instability-high solid tumors	PD-L12	VHH-Fc (Fc IgG1)//Mono	China, 2021		Reduced Fc effector function (ADCC/ADCP)
Belantamab mafodotin-blmf	Multiple Myeloma	BCMA	IgG1/k/Mono	US, 2020	ADC[3]: MMAF[13]	Improved FcγRIIIa binding/Improved ADCC
Elranatamab	Multiple Myeloma	BCMA, CD3	IgG2/k/Bi	US, 2023		Strop Fab-arm exchange method
Teclistamab	Multiple Myeloma	BCMA, CD3	IgG4; Hetero H, HL exchanged/l/Bi	EU, 2022		Hinge-stabilizing (S228P) and Fc-silencing (F234A, L235A)
Daratumumab	Multiple Myeloma	CD38	IgG1/k/Mono	US, 2015		

Isatuximab-irfc	Multiple Myeloma	CD38	IgG1/k/Mono	US, 2020		
Talquetamab-tgvs	Multiple Myeloma	GPCR5D, CD3	IgG4/l, k/Bi	US, 2023		Hinge-stabilization (S228P), Reduced Fc effector function (F234A-L235A)
Elotuzumab	Multiple Myeloma	SLAMF7	IgG1/k/Mono	US, 2015		
Penpulimab	nasopharingeal carcinoma	PD-12	IgG1/n.a./Mono	China, 2021		Reduced Fc effector functions
Toripalimab	nasopharingeal carcinoma	PD-12	IgG4/k/Mono	China, 2018		Hinge stabilizing
Tagitanlimab	nasopharingeal carcinoma	PD-L12	IgG1/k/Mono	in review		Reduced Fc effector function
Mirvetuximab soravtansine	ovarian cancer	FR**α**	IgG1/k/Mono	US, 2022	ADC[3]: DM4[14]	
Olaratumab	sarcoma	PDGFRα	IgG1/k/Mono	US, 2016		
Mogamulizumab-kpkc	Sézary syndrome	CCR4	IgG1/k/Mono	Japan, 2012		Improved FcγRIIIa binding/Improved ADCC
Cosibelimab	squamous cell carcinoma	PD-L12	IgG1/l/Mono	in review		
Cemiplimab-rwlc	squamous cell carcinoma: cutaneous	PD-12	IgG4/k/Mono	US, 2018		Hinge stabilizing
Tislelizumab	squamous cell carcinoma: esophageal	PD-12	IgG4/k/Mono	China, 2019		Hinge stabilizing; Abrogated FcγRs binding
Enfortumab vedotin-ejfv	urothelial	Nectin-4	IgG1/k/Mono	US, 2019	ADC[3]: MMAE[10]	

(a) No INN
(1) n.a. indicates that the data is not available
(2) antibodies targeting immune checkpoint molecules that can be used for the treatment of many tumor types, to switch the host's immune response to generate an anti-tumoral response
(3) ADC or antibody-drug conjugate
(4) RIC is a radioactive isotope conjugated antibody
(5) DXd is an inhibitor of topoisomerase I
(6) DM1 is an inhibitor of tubulin polymerization
(7) Duocarmycin is a DNA minor-groove-binding alkylating agent
(8) Duostatin-5 is a Tubulin inhibitor
(9) SN38 is a topoisomerase I inhibitor
(10) MMAE or Monomethyl Auristatin E is a tubulin polymerization inhibitor
(11) Calicheamicin is DNA-binding and induces double-chain breaks
(12) SG3199 is a cytotoxic DNA minor groove interstrand crosslinking pyrrolobenzodiazepine (PBD) dimer a potent cytotoxic agent for cells
(13) Monomethyl Auristatin F or MMAF is a tubulin polymerization inhibitor
(14) DM4 is an inhibitor of tubulin polymerization
(15) Improved FcγRIIIa binding and decreased affinity for human inhibitory FcγRIIB (CD32B)
(16) Mutations generated in the Ig not yet reported

Table S2. Number of Tumor & Normal samples (N) analyzed in Figures 2B & 3B.

Tissue	Normal	Tumor
Adipose	72	10
Adrenal Gland	501	58
Bladder	65	16
Blood	2623	4694
Bone	14	5
Brain	2569	5296
Breast	567	9867
Cartilage	2	51
Cervix	21	144
Colon	524	4887
Endometrium	73	74
Esophagus	105	328
Eye	1	156
Gallbladder	5	13
Head and Neck	20	217
Heart	41	222
Joint	55	135
Kidney	382	1534
Larynx	2	111
Liver	297	691
Lung	1007	3479
Muscle	684	5
Oral	15	371
Ovary	132	2121
Pancreas	119	365
Pharynx	6	40
Placenta	17	-
Prostate	142	620
Skin	319	1124
Small Intestine	33	12
Soft Tissue	9	149
Stomach	305	1330
Teeth	8	27
Testis	23	188
Thyroid	199	379
Tongue	20	46
Urothelium	9	90
Uterus	113	486
Vagina	5	3
Vulva	14	21
